# Supplementary figures and images for: Cooling the Motion of Diamond Nanocrystals in a Magneto-Gravitational Trap in High Vacuum
Source: Sci Rep. 2016 Jul 22;6:30125. doi: 10.1038/srep30125 (PMC4957077; doi:10.1038/srep30125)

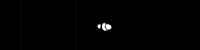

Supplement: Supplementary Information [file srep30125-s1.gif]

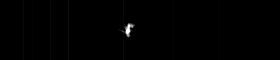

Supplement: Supplementary Information [file srep30125-s3.gif]
