# Supplementary material for: Cooling the Motion of Diamond Nanocrystals in a Magneto-Gravitational Trap in High Vacuum
Source: Sci Rep. 2016 Jul 22;6:30125. doi: 10.1038/srep30125 (PMC4957077; doi:10.1038/srep30125)
Supplement: Supplementary Information [file srep30125-s2.pdf]

Cooling the Motion of Diamond Nanocrystals in a Magneto-Gravitational Trap in High Vacuum: Supplementary Information

Jen-Feng Hsu[1], Peng Ji[1], Charles W. Lewandowski[1], and Brian D'Urso[1,*]

[1]Department of Physics and Astronomy, University of Pittsburgh, Pittsburgh, PA 15260, USA
[*]dursobr@pitt.edu

Multipole Expansion of the Trap Magnetic Scalar Potential

Since there are no currents in the trapping region, the magnetic field can be derived from a magnetic scalar potential Φ_M which must satisfy Laplace's equation,

$$\nabla^2\Phi_M = 0. \tag{1}$$

The general solution for Φ_M can be expanded in spherical harmonics as

$$\Phi_M(r,\theta,\phi) = \sum_{l=0}^{\infty}\sum_{m=-l}^{l}\left[A_{lm}r^l + B_{lm}r^{-(l+1)}\right]Y_l^m(\theta,\phi), \tag{2}$$

where r is the radial distance, θ is the polar angle, and ϕ is the azimuthal, $Y_l^m(\theta,\phi)$ is a complex spherical harmonic, and A_{lm} and B_{lm} are expansion coefficients. For a finite field throughout the trapping region, we must have $B_{lm} = 0$. Based on the trap symmetry, the potential profile of the trap, and the axes designations (x for the transverse direction, y for the vertical direction, and z for the axial direction), three real spherical harmonics are chosen to approximate the potential. Expressed in Cartesian coordinates, they are:

$$Y_{2,-2}(x,y,z) = i\sqrt{\frac{1}{2}}\left(Y_2^{-2}(x,y,z) - Y_2^{2}(x,y,z)\right) = \frac{1}{2}\sqrt{\frac{15}{\pi}}\frac{xy}{r^2} \tag{3}$$

$$Y_{3,1}(x,y,z) = \sqrt{\frac{1}{2}}\left(Y_3^{-1}(x,y,z) - Y_3^{1}(x,y,z)\right) = \frac{1}{4}\sqrt{\frac{21}{2\pi}}\frac{x(4z^2-x^2-y^2)}{r^3} \tag{4}$$

$$Y_{4,-4}(x,y,z) = i\sqrt{\frac{1}{2}}\left(Y_4^{-4}(x,y,z) - Y_4^{4}(x,y,z)\right) = \frac{3}{4}\sqrt{\frac{35}{\pi}}\frac{xy(x^2-y^2)}{r^4}. \tag{5}$$

Each real spherical harmonic contributes a corresponding term to the magnetic scalar potential:

$$\Phi_{2,-2}(x,y,z) = \left(\frac{a_2y_0}{2}\right)\left(\frac{1}{y_0}\right)^2 r^2 Y_{2,-2}(x,y,z) \tag{6}$$

$$\Phi_{31}(x,y,z) = \left(\frac{a_3y_0}{3}\right)\left(\frac{1}{y_0}\right)^3 r^3 Y_{3,1}(x,y,z) \tag{7}$$

$$\Phi_{4,-4}(x,y,z) = \left(\frac{a_4y_0}{4}\right)\left(\frac{1}{y_0}\right)^4 r^4 Y_{4,-4}(x,y,z), \tag{8}$$

where a_2, a_4, and a_3 are the coefficients to be solved using the observed centre-of-mass oscillation frequencies, and y_0 is a length scale, chosen to be the vertical distance from the geometrical centre of the trap to the upper or lower pole pieces. The arrangement of the factors in front of each term is such that a_2, a_4, and a_3 will be in units of Tesla. The magnetic scalar potential Φ_M can then be used to calculate the magnetic field as in the main text.

Trajectory Tracking

A microscope slide with tick marks at 10 µm intervals is imaged onto the high-speed camera through the same optical path as used for imaging trapped particles. This results in a calibration of 0.259 µm per pixel.

The high speed camera images are analysed using trackpy, which determines the position of the particle in each frame. In approximately 1% of the frames, the detected light is too dim for trackpy to locate the particle. In these cases, we replace the location of the particle with the average location of the particle in all frames where the particle could be located. Increasing the threshold brightness for finding the particle such that the number of frames rejected increases by a factor of two or more has only a small impact on the reported results; in particular the measured cooled temperature of the centre-of-mass motion appears to be biased up by the loss of data.

Trapped Particle in Motion

Supplementary video V1 is an animated .gif file of the high-speed images at 0.053 mbar. Supplementary video V2 shows the cooled motion at high vacuum. The frame rate in the videos has been slowed by a factor of ten to improve visibility of the motion.

Centre-of-Mass Motion Data

Supplementary Table S1 summarises the full centre-of-mass motion results from one diamond nanocrystal cluster at several pressures. Statistical uncertainties are given for each measurement, which are calculated from the standard deviation of each PSD point over 30 trials. The mass of the particle m and the oscillation frequencies (ω_x, ω_y, and ω_z) reported in the main text are weighted averages of the values calculated at rough vacuum and ambient temperature, but the final uncertainties are dominated by the systematic error which can be seen in the variation of those values with background gas pressure. The cooled temperature uncertainties are also larger than the purely statistical errors in the table due to systematic uncertainty in m.

Pressure (mbar)	Direction	f_0 (Hz)	Γ_{f0} (Hz)	S_0 (μm^2)	m (pg)	T (K)
5.3×10^{-2}	Axial	9.64 ± 0.02	3.39 ± 0.05	24.5 ± 0.3	28.8 ± 0.4	assumed 295
5.3×10^{-2}	Vertical	129.53 ± 0.02	3.56 ± 0.06	0.151 ± 0.002	25.9 ± 0.3	assumed 295
5.3×10^{-2}	Transverse	104.03 ± 0.04	3.2 ± 0.1	n/a	n/a	assumed 295
2.7×10^{-2}	Axial	9.56 ± 0.01	1.67 ± 0.03	23.3 ± 0.3	30.8 ± 0.4	assumed 295
2.7×10^{-2}	Vertical	129.56 ± 0.02	1.75 ± 0.04	0.146 ± 0.002	26.9 ± 0.4	assumed 295
2.7×10^{-2}	Transverse	104.03 ± 0.02	1.66 ± 0.05	n/a	n/a	assumed 295
1.3×10^{-2}	Axial	9.58 ± 0.01	0.87 ± 0.02	23.5 ± 0.5	30.5 ± 0.6	assumed 295
1.3×10^{-2}	Vertical	129.65 ± 0.01	0.91 ± 0.03	0.149 ± 0.004	26.3 ± 0.7	assumed 295
1.3×10^{-2}	Transverse	104.05 ± 0.01	0.87 ± 0.02	n/a	n/a	assumed 295
6.7×10^{-3}	Axial	9.57 ± 0.01	0.40 ± 0.01	25.9 ± 0.7	27.7 ± 0.7	assumed 295
6.7×10^{-3}	Vertical	129.66 ± 0.01	0.47 ± 0.02	0.150 ± 0.006	26 ± 1	assumed 295
6.7×10^{-3}	Transverse	104.10 ± 0.01	0.44 ± 0.02	n/a	n/a	assumed 295
HV cooled	Axial	10.3 ± 0.2	10.6 ± 0.6	0.046 ± 0.003	assumed 27.8	0.60 ± 0.05
HV cooled	Vertical	130.72 ± 0.08	6.3 ± 0.3	$(1.5\pm0.1)\times10^{-3}$	assumed 27.8	3.2 ± 0.2
HV cooled	Transverse	105.00 ± 0.01	n/a	n/a	n/a	n/a

Supplementary Table S1. Details of centre-of-mass motion for a single diamond nanocrystal cluster. High vacuum (HV) indicates a pressure of 7×10^{-8} mbar. All reported uncertainties are statistical.
